# Family satisfaction in an interdisciplinary ICU: a quality audit

**DOI:** 10.1186/cc11103

**Published:** 2012-03-20

**Authors:** UM Schmid, R Alpiger, T Rizzo, CK Hofer

**Affiliations:** 1 Triemli City Hospital, Zurich, Switzerland

## Introduction

The quality of intensive care medicine depends on multiple indicators [1,2]. Meeting relatives' needs in the challenging situation of ICU visits is crucial. The aim of this audit was to assess next of kin's satisfaction and influencing factors.

## Methods

With institutional approval, questionnaires were distributed to family members of ICU patients. The survey included two visual analogue scale ratings (VAS 1: patient care, VAS 2: decision-making) and 24 questions with four dimensions D1 to D4 (general impression, treatment and patient care quality, professional quality) on a five-point Likert scale, transformed into values 1 to 100. Patient-specific and relatives' sociodemographic data were recorded. Data are presented as the mean ± SD, median (Q.5), interquartile range (IQR) and range (minimum/maximum). Subgroup analysis (relative's and patient's age, sex, education, marital status, length of stay, visit frequency and mortality) was performed using the Mann-Whitney U test.

## Results

Questionnaires of 159 patients were analyzed (patients: age = 66.1 ± 13.0 years, 64% female, SAPS = 38.8 ± 17.5, LOS = 13.5 ± 11.8 days, mortality = 16.5%; relatives: age = 44.5 ± 26.9 years, 63.7% female, 13% medical/25.5% higher education). High satisfaction (VAS 1/2, D1 to D4) was observed (Table 1). Significant differences within subgroups were found: relatives with healthcare education showed higher D1 to D4 satisfaction than the ones with a graduate degree only. Higher VAS scorings were observed from next of kin with high visit frequency (≥5×/week).

**Table 1:** 

	Mean ± SD	Q.5/IQR	Minimum/maximum
VAS1	9.1 ± 0.9	9.3/0.9	5.5/10
VAS2	8.6 ± 1.5	9.0/1.5	3.1/10
∑Satisf	87 ± 15.1	80/20	20/100
D1	91.1 ± 15	100/20	20/100
D2	89.2 ± 13	100/20	40/100
D3	86 ± 15.4	80/20	20/100
D4	85.5 ± 15	80/40	20/100

## Conclusion

Relatives of ICU patients were in general highly satisfied. The educational status and ICU visit frequency of the next of kin were revealed to be influencing factors.
